# Exploratory Toxicogenomic Profiling Identifies Candidate DINCH-Responsive Genes Relevant to Prostate Cancer

**DOI:** 10.3390/ijms27177686

**Published:** 2026-08-27

**Authors:** Chi-Fen Chang, Wen-Hsin Lin, Chao-Yuan Huang, Chia-Cheng Yu, Victor C. Lin, Te-Ling Lu, Shu-Pin Huang, Bo-Ying Bao

**Affiliations:** 1 Department of Anatomy, School of Medicine, China Medical University, Taichung 406, Taiwan; cfchang@mail.cmu.edu.tw; 2 Department of Pharmacy, China Medical University, Taichung 406, Taiwan; wslin@mail.cmu.edu.tw (W.-H.L.); lutl@mail.cmu.edu.tw (T.-L.L.); 3 Department of Urology, National Taiwan University Hospital, College of Medicine, National Taiwan University, Taipei 100, Taiwan; cyh0909@ntu.edu.tw; 4 Division of Urology, Department of Surgery, Kaohsiung Veterans General Hospital, Kaohsiung 813, Taiwan; ccyu@vghks.gov.tw; 5 Department of Urology, School of Medicine, National Yang Ming Chiao Tung University, Taipei 112, Taiwan; 6 Department of Pharmacy, College of Pharmacy and Health Care, Tajen University, Pingtung 907, Taiwan; 7 Department of Urology, E-Da Hospital, Kaohsiung 824, Taiwan; ed102161@edah.org.tw; 8 School of Medicine for International Students, I-Shou University, Kaohsiung 840, Taiwan; 9 Graduate Institute of Clinical Medicine, College of Medicine, Kaohsiung Medical University, Kaohsiung 807, Taiwan; 10 Department of Urology, Kaohsiung Medical University Hospital, Kaohsiung 807, Taiwan; 11 Institute of Medical Science and Technology, College of Medicine, National Sun Yat-Sen University, Kaohsiung 804, Taiwan

**Keywords:** plasticizer, DINCH, prostate cancer, transcriptomics, toxicogenomics

## Abstract

Diisononyl cyclohexane-1,2-dicarboxylate (DINCH), a non-phthalate plasticizer adopted as a safer alternative for food-contact and medical-grade materials, is ubiquitously detected in human biomonitoring studies. Despite widespread exposure, its transcriptional effects in prostate cells and the potential prostate cancer relevance of DINCH-responsive genes remain unclear. We integrated transcriptomic profiling of DINCH-exposed human prostate epithelial cells with exploratory genetic association analyses in 630 patients with prostate cancer receiving androgen deprivation therapy (ADT). Haplotype-tagged single-nucleotide polymorphisms (SNPs) in candidate DINCH-responsive genes were evaluated for their association with overall survival (OS) and cancer-specific survival (CSS). The prostate cancer relevance of the prioritized genes was further validated using pooled multi-cohort bioinformatic analyses. DINCH exposure produced an exploratory molecular signature comprising 83 genes across all tested doses, broadly suppressing cell–matrix adhesion pathways and activating chromatin remodeling. Exploratory genetic screening identified nominal associations of *LPP* rs1040033 with OS (*p* = 0.0002, *q* = 0.131) and *FAM111B* rs7110278 with CSS (*p* = 0.0010, *q* = 0.575); neither association remained significant after multiple-testing correction. DINCH exposure significantly downregulated *LPP* and upregulated *FAM111B* expression in prostate epithelial cells. Independently, pooled analyses demonstrated reduced *LPP* and elevated *FAM111B* expression in prostate cancer tissues compared with normal prostate tissues. Higher *LPP* expression predicted a favorable prognosis, whereas elevated *FAM111B* predicted worse survival. Pathway analyses linked low *LPP* expression to impaired adhesion signaling and metabolic reprogramming, whereas high *FAM111B* expression was associated with mitotic and cell-cycle activation. DINCH exposure induced exploratory transcriptional alterations involving *LPP*-related adhesion pathways and *FAM111B*-related proliferative signaling. The genetic findings are exploratory and require independent validation. Although public datasets support the prognostic relevance of *LPP* and *FAM111B* in prostate cancer, they do not link these genes to DINCH exposure.

## 1. Introduction

Plasticizers are chemical additives widely incorporated into polyvinyl chloride-based materials to enhance their flexibility and durability, resulting in pervasive human exposure through food packaging, medical devices, and consumer products. Phthalate-based plasticizers, particularly di-2-ethylhexyl phthalate (DEHP) and diisononyl phthalate (DINP), have dominated industrial applications for decades; however, accumulating evidence of their endocrine-disrupting, reproductive, and other adverse biological effects has prompted regulatory restrictions worldwide and has accelerated the adoption of structurally distinct alternatives [1]. In response to these restrictions, manufacturers have increasingly replaced DEHP with alternative plasticizers, including di-2-ethylhexyl terephthalate (DEHTP), acetyl tributyl citrate, and diisononyl cyclohexane-1,2-dicarboxylate (DINCH), leading to rapidly changing patterns of human exposure globally [2]. Although these compounds were introduced as safer alternatives, growing toxicological evidence suggests that several replacement plasticizers may exhibit biological activities overlapping those of legacy phthalates [3]. Similar to DEHP and DINP, DINCH and its metabolites have been shown to interact with endocrine-related signaling pathways, including estrogen receptor, androgen receptor (AR), and peroxisome proliferator-activated receptor (PPAR) signaling, indicating the capacity to influence hormone-regulated physiological processes [4]. Comparative experimental studies further demonstrate that both traditional phthalates (DEHP and DINP) and newer substitutes such as DINCH and DEHTP can disrupt steroidogenesis and endocrine homeostasis, although their relative potencies and modes of action may differ [3]. Moreover, developmental and metabolic studies have reported that DINCH exposure can induce oxidative stress, inflammatory responses, lipid metabolic disturbances, and reproductive alterations, supporting the growing view that replacement plasticizers should be evaluated with the same rigor as the phthalates they are intended to replace [5,6]. Diisononyl cyclohexane-1,2-dicarboxylate (DINCH) was developed as a non-phthalate replacement and approved for use in sensitive applications, including food-contact packaging, children’s toys, and medical devices intended for vulnerable populations such as neonates and patients undergoing dialysis [2,7]. The use of DINCH has consequently expanded rapidly, and its primary urinary metabolites, cyclohexane-1,2-dicarboxylic acid monohydroxyisononyl ester and cyclohexane-1,2-dicarboxylic acid, have now been consistently detected in population biomonitoring studies across multiple continents, confirming its widespread and increasing human exposure [8,9].

Despite its initially favorable regulatory profile, emerging evidence has challenged the assumption that DINCH is biologically inert. In vitro studies have demonstrated that DINCH and its metabolites activate PPAR family members at environmentally relevant concentrations by engaging the same nuclear receptor axes implicated in phthalate-mediated endocrine disruption [4]. DINCH metabolites further perturb steroid hormone biosynthesis, disrupt thyroid hormone homeostasis, and modify lipid metabolic programs across diverse experimental systems [10,11]. Epidemiological studies have reported associations between urinary DINCH metabolite concentrations and markers of altered reproductive hormone levels and oxidative stress in human cohorts [12,13]. Collectively, these observations raise substantive concerns that the regulatory transition from phthalates to DINCH may not fully eliminate endocrine-disrupting risks.

The relationship between endocrine-disrupting chemical exposure and prostate cancer is particularly plausible, given the profound hormonal sensitivity of the prostate gland. Prostate cancer is the most frequently diagnosed non-cutaneous malignancy in men worldwide, and androgen deprivation therapy (ADT) remains the cornerstone of management for advanced disease [14]. Nevertheless, virtually all patients ultimately develop castration resistance, underscoring the clinical importance of identifying environmental and genetic modifiers that influence progression under hormonal suppression. Prior investigations have established that endocrine-disrupting chemicals, including phthalate metabolites, modulate AR transcriptional activity, induce oxidative stress, and reprogram epithelial cell adhesion programs in prostate model systems [15,16]. Although data directly linking DINCH to prostate cancer outcomes remain absent, its mechanistic parallels with established endocrine disruptors and the rapidly expanding scale of human exposure provide a compelling rationale for investigation.

Previous investigations of phthalate-associated compounds have shown that inherited variation in *PPAR* genes and enzymes involved in xenobiotic metabolism can influence susceptibility to hormone-dependent cancers, providing a biological basis for exploring integrative toxicogenomic approaches in the context of DINCH exposure [17,18,19]. Endocrine-disrupting plasticizers may also perturb extracellular matrix signaling and epithelial organization, thereby linking hormonal dysregulation to cellular processes relevant to cancer progression. Notably, the bioactive phthalate metabolite MEHP suppresses focal adhesion, cell–matrix interaction, and cell-junction pathways in human prostate epithelial cells, suggesting that plasticizer-induced endocrine perturbation may converge with adhesion remodeling to compromise epithelial integrity [15]. Building on this concept, we combined transcriptomic analysis of DINCH-treated human prostate epithelial cells with a genetic association study involving 630 patients with prostate cancer undergoing ADT. Although our research group has previously used this ADT-treated cohort to investigate other candidate genes associated with prostate cancer progression, the present study addresses a distinct biological hypothesis by employing an integrated toxicogenomic discovery strategy. Rather than evaluating predefined candidate genes, we first identified an exploratory set of DINCH-responsive genes through transcriptomic profiling and subsequently prioritized clinically relevant candidates using genetic association analyses integrated with independent transcriptomic and survival analyses. Because direct personal DINCH exposure status and germline genotypes were not measured within the same patient cohort, this study applies an integrative multi-stage discovery strategy to identify candidate DINCH-responsive genes and explore their independent prognostic relevance in prostate cancer.

## 2. Results

**Figure 1:**
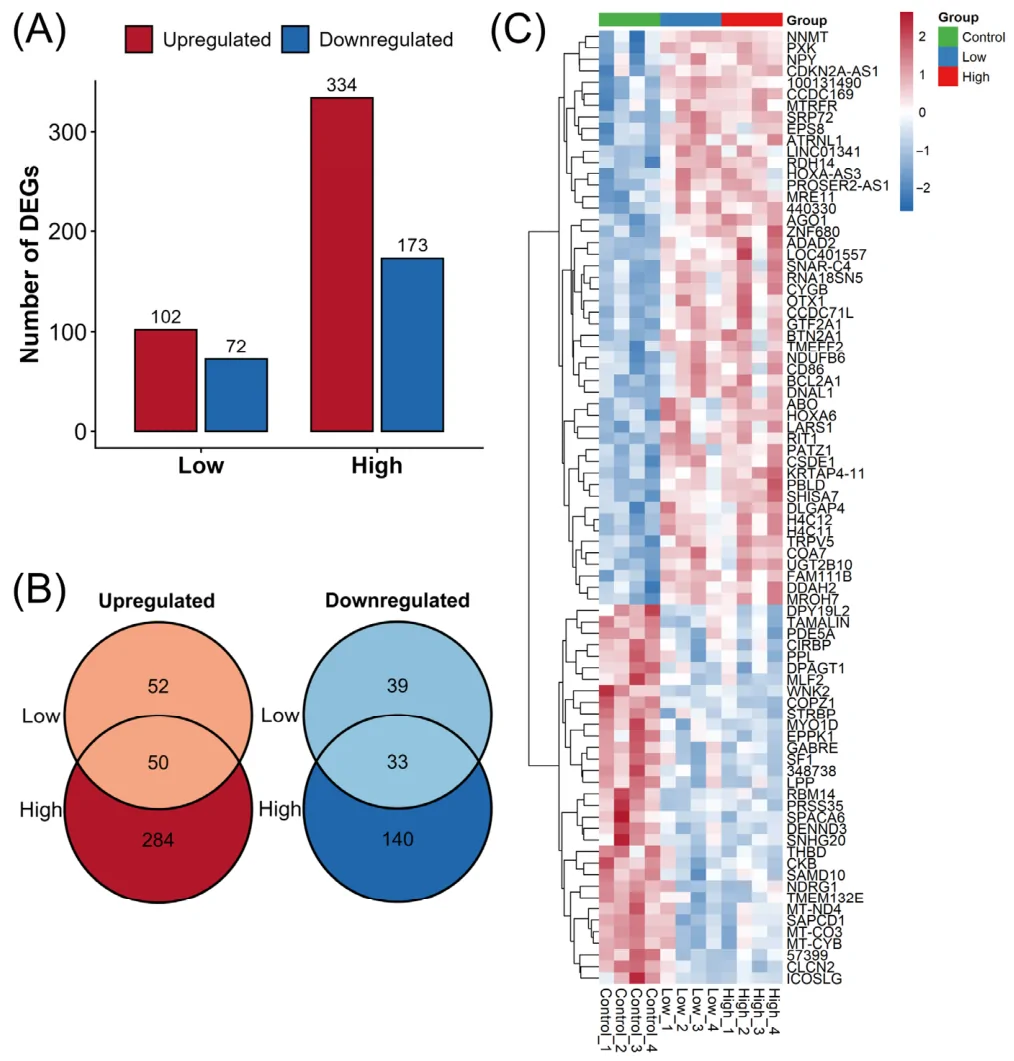
Transcriptomic profiling and differential expression analysis of human prostate epithelial cells exposed to diisononyl cyclohexane-1,2-dicarboxylate (DINCH). (**A**) Total number and direction of regulation for differentially expressed genes (DEGs; *p* < 0.01) identified under low-dose (0.01 μM) and high-dose (1 μM) DINCH treatments relative to dimethyl sulfoxide vehicle controls in immortalized normal human prostatic epithelial PNT1A cells. (**B**) Venn diagrams illustrating the intersection of shared upregulated and downregulated DEGs between the low- and high-dose exposure groups. (**C**) Hierarchical clustering and heatmap visualization of the top DEGs across biological replicates, stratified into control, low-dose, and high-dose treatment groups. The color scale indicates relative gene expression levels (Z-score-normalized), ranging from downregulated (blue) to upregulated (red).

To characterize the transcriptional consequences of DINCH exposure in prostate epithelial cells, immortalized normal human prostatic epithelial PNT1A cells were treated with vehicle control (dimethyl sulfoxide, DMSO) and low-dose (0.01 μM) or high-dose (1 μM) DINCH for 48 h. Differential expression analysis at a nominal threshold of *p* < 0.01 identified 174 differentially expressed genes (DEGs) (102 upregulated and 72 downregulated) under low-dose exposure and 507 DEGs (334 upregulated and 173 downregulated) under high-dose exposure; however, none remained significant after false discovery rate (FDR) correction (minimum *q*-value = 0.323), with the higher tested concentration eliciting a broader transcriptional response than the lower concentration (Figure 1A). However, because only two concentrations were evaluated, these findings should not be interpreted as evidence of a dose–response relationship. Comparative analysis of the two exposure conditions using Venn diagrams revealed that 50 genes were shared among the upregulated DEGs and 33 were shared among the downregulated DEGs, totaling 83 genes concordantly altered across both concentrations (Figure 1B); these genes represent an exploratory candidate signature rather than a definitive list of FDR-significant DEGs. Hierarchical clustering and heatmap visualization of these shared DEGs confirmed that replicate biological samples exhibited coherent directional expression patterns relative to vehicle-treated controls (Figure 1C and Table S2).

**Figure 2:**
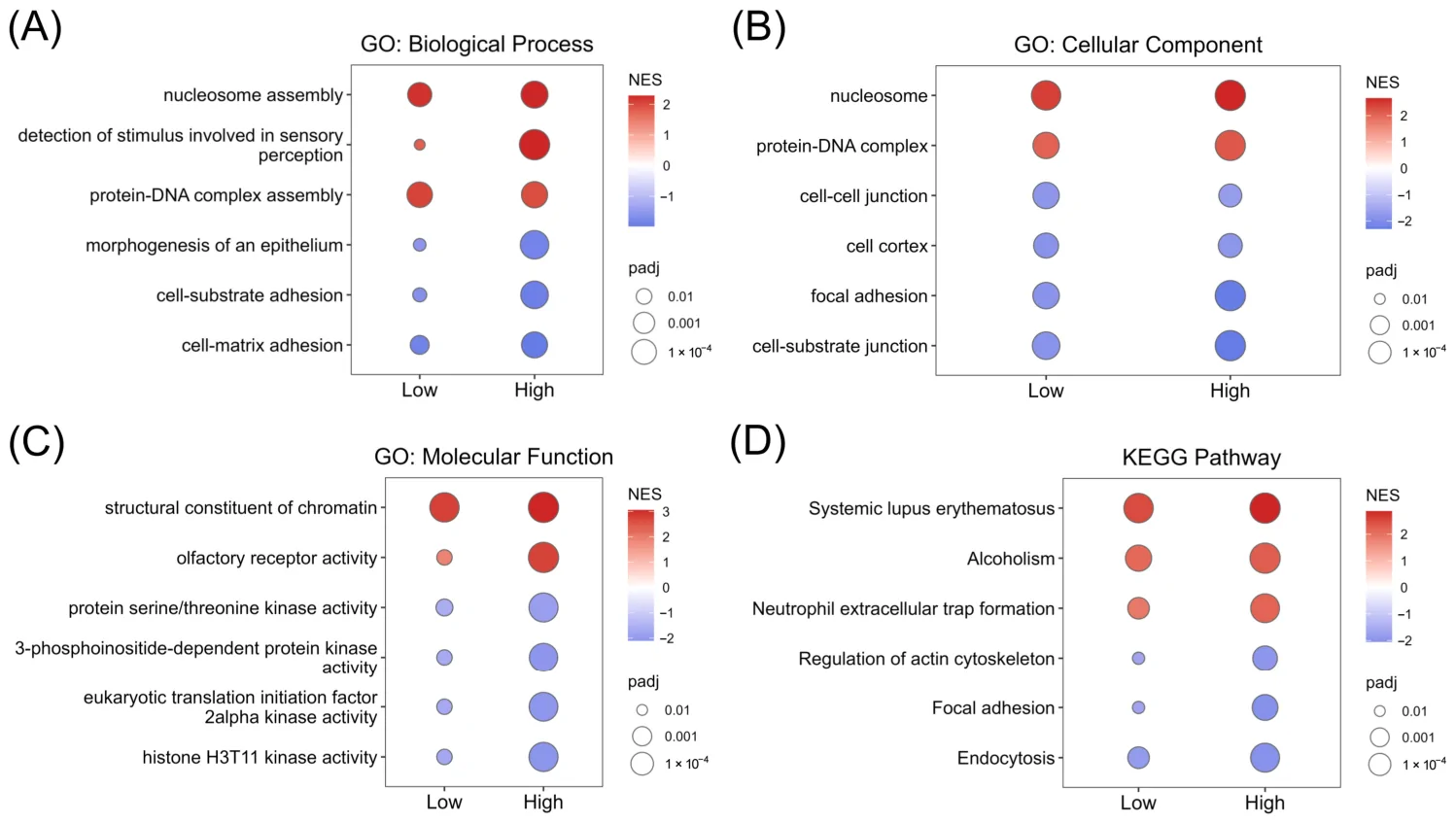
Functional enrichment and pathway analysis of DINCH-responsive genes. Gene Set Enrichment Analysis (GSEA) dot plots demonstrating significantly altered molecular axes following low- and high-dose DINCH exposure. The enriched terms were categorized according to the Gene Ontology (GO) as follows: (**A**) biological processes, (**B**) cellular components, (**C**) molecular functions, and (**D**) Kyoto Encyclopedia of Genes and Genomes (KEGG) pathways. The color gradient corresponds to the normalized enrichment score (NES), while the bubble size reflects the adjusted *p*-value (*p*adj).

To elucidate the biological significance of DINCH-induced transcriptional changes, we performed gene set enrichment analysis (GSEA) using ranked differential expression profiles for Gene Ontology (GO) biological processes, cellular components, molecular functions, and Kyoto Encyclopedia of Genes and Genomes (KEGG) pathways. Biological process analysis revealed that terms related to nucleosome assembly and protein–DNA complex assembly were among the most positively enriched (elevated normalized enrichment score) under both low- and high-dose conditions, whereas cell–substrate adhesion and cell–matrix adhesion were negatively enriched, indicating the suppression of adhesion-related programs following in vitro exposure to DINCH (Figure 2A). Consistent with these findings, cellular component analysis demonstrated positive enrichment of nucleosome and protein–DNA complex terms, along with marked negative enrichment of focal adhesion and cell–substrate junction components (Figure 2B). Molecular function analysis further highlighted the upregulation of structural constituents of chromatin, whereas histone H3T11 kinase activity and protein serine/threonine kinase activity were negatively enriched (Figure 2C). KEGG pathway analysis corroborated these observations, revealing positive enrichment of immune-related pathways, including systemic lupus erythematosus, alcoholism, and neutrophil extracellular trap formation, and significant negative enrichment of focal adhesion, actin cytoskeleton regulation, and endocytosis pathways at both DINCH concentrations (Figure 2D). Collectively, these pathway-level alterations demonstrate that DINCH exposure broadly perturbs cell–matrix adhesion while simultaneously activating chromatin remodeling pathways in cultured prostate epithelial cells.

**Figure 3:**
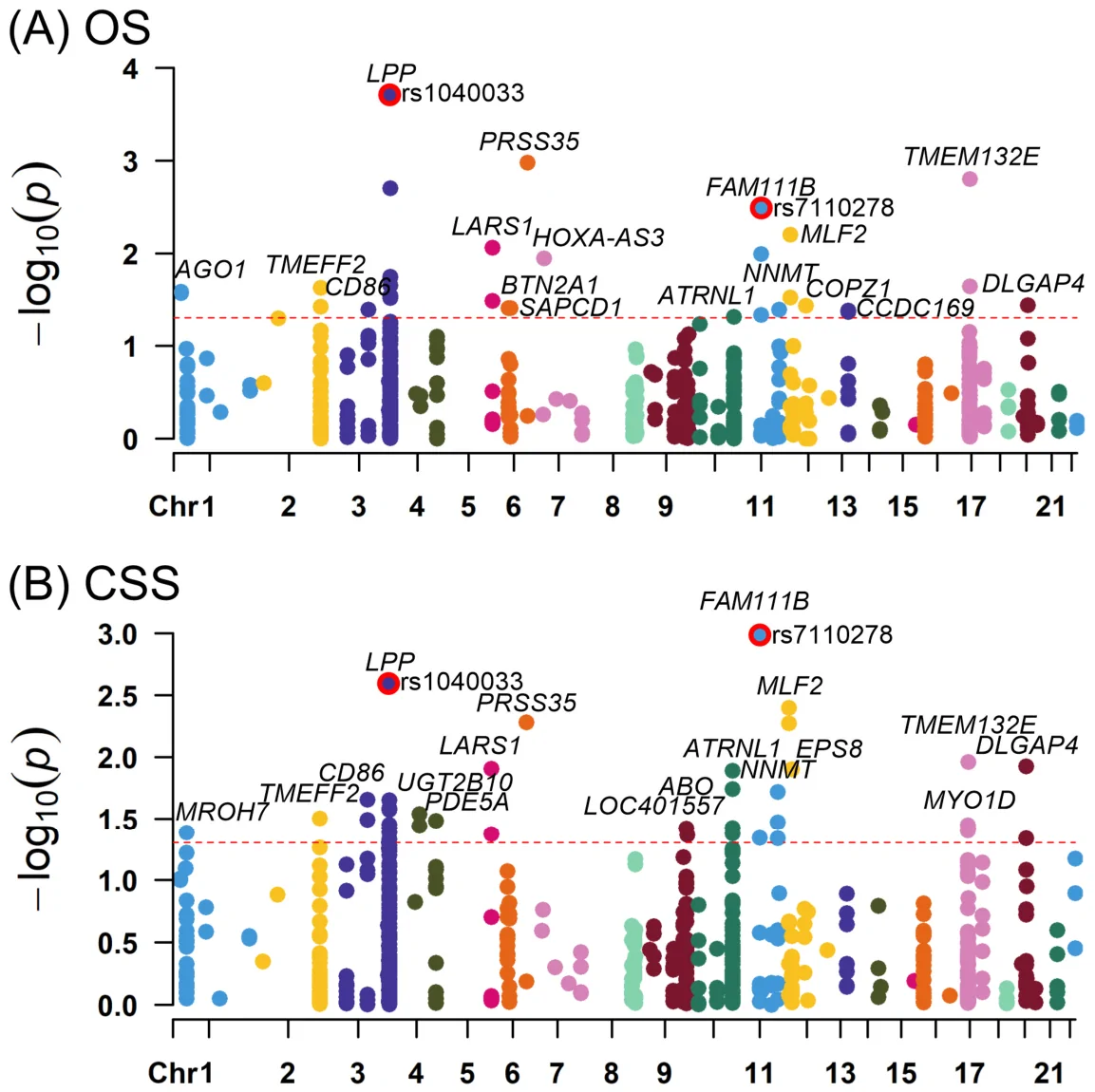
Association of single-nucleotide polymorphisms (SNPs) in DINCH-regulated genes with clinical outcomes in patients with prostate cancer. Manhattan plots mapping the prognostic significance of germline variants across candidate genes for (**A**) overall survival (OS) and (**B**) prostate cancer-specific survival (CSS) in a clinical cohort of patients undergoing androgen deprivation therapy (ADT). The *y*-axis displays the −log10(*p*) values across chromosomal locations (*x*-axis). The dashed red line denotes the nominal significance threshold (*p* = 0.05), with prominent candidate genes highlighted and annotated by SNPs showing the strongest nominal associations (e.g., rs1040033 in *LPP* and rs7110278 in *FAM111B*).

To investigate whether germline variations within DINCH-regulated genes correlate with prostate cancer outcomes, 666 quality-controlled haplotype-tagged single-nucleotide polymorphisms (SNPs) spanning 69 shared DEGs were genotyped in an independent cohort of 630 patients with prostate cancer receiving ADT and evaluated for associations with overall survival (OS) and cancer-specific survival (CSS). Manhattan plots of the association results revealed that multiple SNPs surpassed the nominal screening threshold (*p* < 0.05) for both clinical endpoints; however, these signals were interpreted as exploratory because multiple SNPs and endpoints were tested. For OS, the most prominently associated signal arose from *LPP* rs1040033 (Figure 3A); carriers of the minor T allele exhibited a nominally increased risk of all-cause mortality compared with carriers of the major G allele (hazard ratio [HR] = 1.32, 95% confidence interval [CI] = 1.14–1.52, *p* = 0.0002, although this association did not remain significant after FDR correction *q* = 0.131). For CSS, *FAM111B* rs7110278 emerged as the most significant association (Figure 3B); carriers of the minor G allele exhibited an increased risk of prostate cancer-specific mortality compared with carriers of the major A allele (HR = 1.31, 95% CI = 1.12–1.54, *p* = 0.0010, but this association was not significant after FDR correction *q* = 0.575). *LPP* rs1040033 ranked as the second most significant CSS association, with carriers of the minor T allele likewise demonstrating an increased risk of prostate cancer-specific mortality compared with carriers of the major G allele (HR = 1.29, 95% CI = 1.09–1.52, *p* = 0.0025, *q* = 0.575). In sensitivity analyses adjusting for age, prostate-specific antigen (PSA) level at ADT initiation, clinical stage, and Gleason score, the associations remained consistent. Specifically, *LPP* rs1040033 remained associated with OS (adjusted HR = 1.28, 95% CI = 1.10–1.48, *p* = 0.0013), whereas *FAM111B* rs7110278 remained associated with CSS (adjusted HR = 1.29, 95% CI = 1.09–1.53, *p* = 0.0026). These adjusted analyses were performed as sensitivity analyses and did not alter the exploratory interpretation of the genetic findings. Both prioritized variants satisfied the prespecified genotyping quality-control criteria and showed no evidence of deviation from Hardy–Weinberg equilibrium (HWE), with *p*-values of 0.651 for *LPP* rs1040033 and 0.860 for *FAM111B* rs7110278. Because neither rs1040033 nor rs7110278 reached statistical significance after multiple-testing correction, they represent suggestive signals rather than confirmed prognostic markers, though their shared identification across endpoints served as a heuristic strategy to prioritize candidate loci for further biological contextualization.

**Figure 4:**
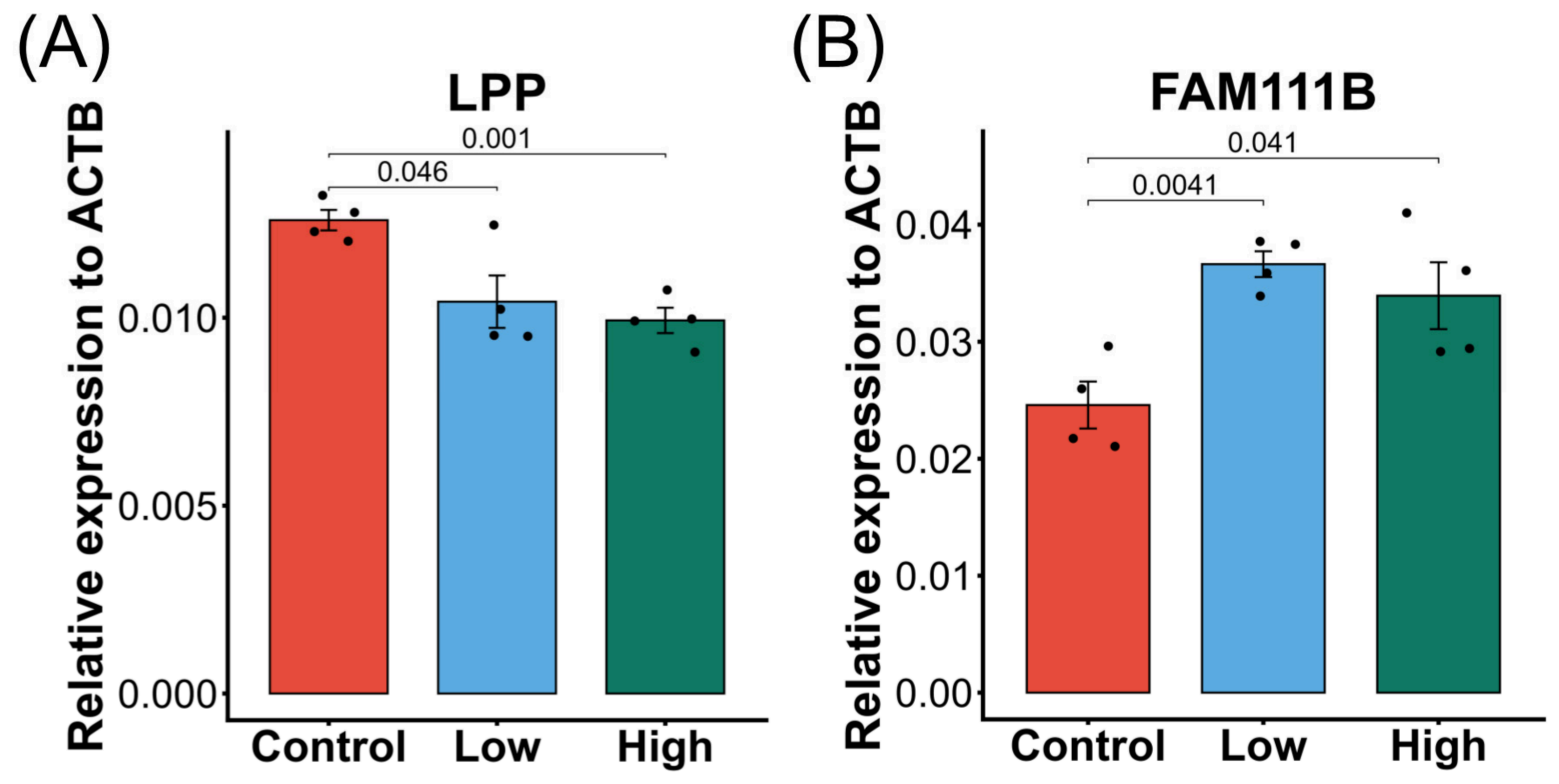
Expression profiles of key DINCH-responsive genes identified from genetic association analysis. Relative mRNA expression levels of (**A**) lipoma-preferred partner (*LPP*) and (**B**) family with sequence similarity 111 member B (*FAM111B*), normalized against β-actin (*ACTB*), across vehicle control, low-dose, and high-dose DINCH treatment groups in PNT1A cells. Data are presented as mean ± standard deviation. Statistically significant differences between groups are indicated by horizontal brackets with the corresponding raw *p*-values.

To further characterize the expression responses of the prioritized candidate genes to DINCH exposure, we quantified the relative mRNA expression of *LPP* and *FAM111B*—normalized to that of *ACTB*—in PNT1A cells across the vehicle control, low-dose, and high-dose DINCH treatment groups. *LPP* expression was significantly reduced in both the low-dose (*p* = 0.046) and high-dose (*p* = 0.001) DINCH treatment groups relative to that in the vehicle controls (Figure 4A). In contrast, *FAM111B* expression was significantly elevated following low-dose (*p* = 0.0041) and high-dose (*p* = 0.041) DINCH treatment compared to that in controls (Figure 4B).

**Figure 5:**
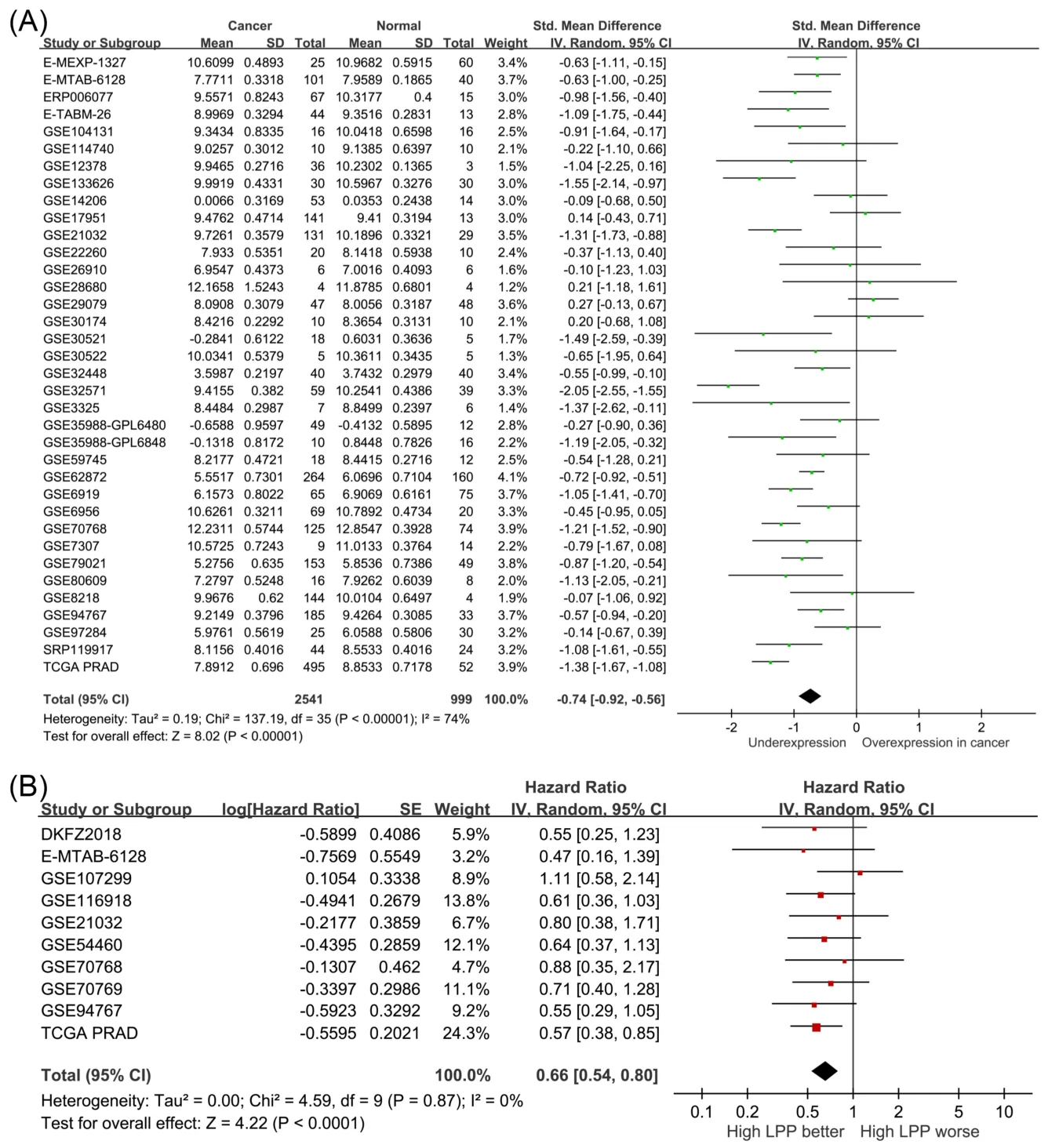
Clinical significance of *LPP* expression in prostate cancer. (**A**) Pooled analysis of public datasets showing significantly reduced *LPP* expression levels in prostate cancer tissues compared with normal prostate tissues. Green squares represent point estimates (standardized mean difference [SMD]) for individual studies, with the square size proportional to the study’s statistical weight. Horizontal lines denote 95% confidence intervals (CIs). Black diamonds represent the overall pooled effect size, with their width corresponding to the overall 95% CI. (**B**) Pooled survival analysis demonstrating that high *LPP* expression was significantly associated with improved prognosis in patients with prostate cancer. Red squares represent point estimates (hazard ratios [HRs]) for each study. SD, standard deviation; SE, standard error; IV, inverse variance; Std, standardized; TCGA PRAD, The Cancer Genome Atlas Prostate Adenocarcinoma; df, degrees of freedom.

To determine the pathological and prognostic significance of *LPP* and *FAM111B* expression in prostate cancer independent of toxicant exposure, we conducted pooled analyses using publicly available prostate cancer transcriptomic datasets. Across 36 independent cohorts comprising 2541 prostate cancer specimens and 999 normal prostate tissues, *LPP* expression was consistently reduced in tumor samples relative to normal controls (standardized mean difference [SMD] = −0.74, 95% CI = −0.92 to −0.56, *p* < 0.0001; Figure 5A). Although between-study heterogeneity was substantial (*I*^2^ = 74%), the directional finding of reduced *LPP* expression in cancer was consistent across most contributing cohorts. Complementary pooled survival analysis across 10 independent studies revealed that higher *LPP* expression was significantly associated with improved patient prognosis (HR = 0.66, 95% CI = 0.54–0.80, *p* < 0.0001; Figure 5B), with negligible between-study heterogeneity (*I*^2^ = 0%). These multi-cohort data establish the clinical and prognostic relevance of *LPP* expression in prostate tumor biology, paralleling the transcriptional downregulation observed following in vitro DINCH exposure.

**Figure 6:**
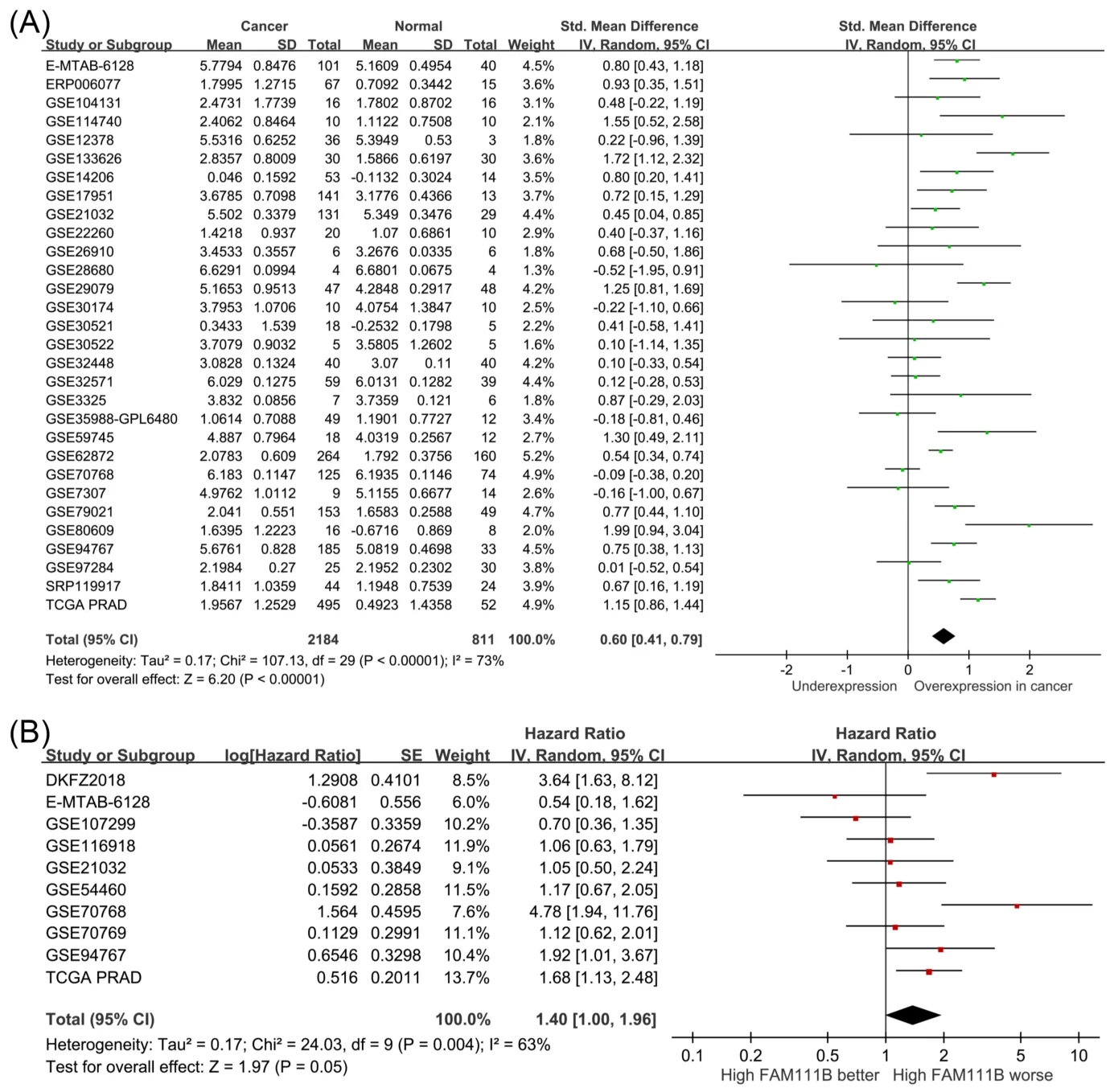
Clinical significance of *FAM111B* expression in prostate cancer. (**A**) Forest plot profiling the standardized mean difference (SMD) in *FAM111B* transcript levels across multiple independent cohorts, comparing prostate cancer samples with normal prostate tissues. (**B**) Consolidated forest plot displaying the hazard ratios (HRs) for survival according to *FAM111B* expression. SD, standard deviation; SE, standard error; IV, inverse variance; Std, standardized; TCGA PRAD, The Cancer Genome Atlas Prostate Adenocarcinoma; df, degrees of freedom.

In direct contrast, a pooled analysis of 30 independent cohorts comprising 2184 cancer and 811 normal tissue samples revealed that *FAM111B* expression was significantly higher in prostate cancer than in normal prostate tissues (SMD = 0.60, 95% CI = 0.41–0.79, *p* < 0.0001; Figure 6A), with moderate-to-high between-study heterogeneity (*I*^2^ = 73%). Pooled survival analysis across 10 independent studies demonstrated that higher *FAM111B* expression was significantly associated with worse patient prognosis (HR = 1.40, 95% CI = 1.00–1.96, *p* = 0.0485; Figure 6B), with moderate cross-cohort variance (*I*^2^ = 63%). These findings demonstrate that *FAM111B* is overexpressed in malignant prostate tissue and associated with unfavorable clinical prognosis, establishing its disease relevance independently of plasticizer exposure. Because the public transcriptomic cohorts did not include DINCH exposure measurements, these pooled analyses evaluate the general prostate cancer relevance of *LPP* and *FAM111B* expression rather than DINCH-specific expression effects in patients.

**Figure 7:**
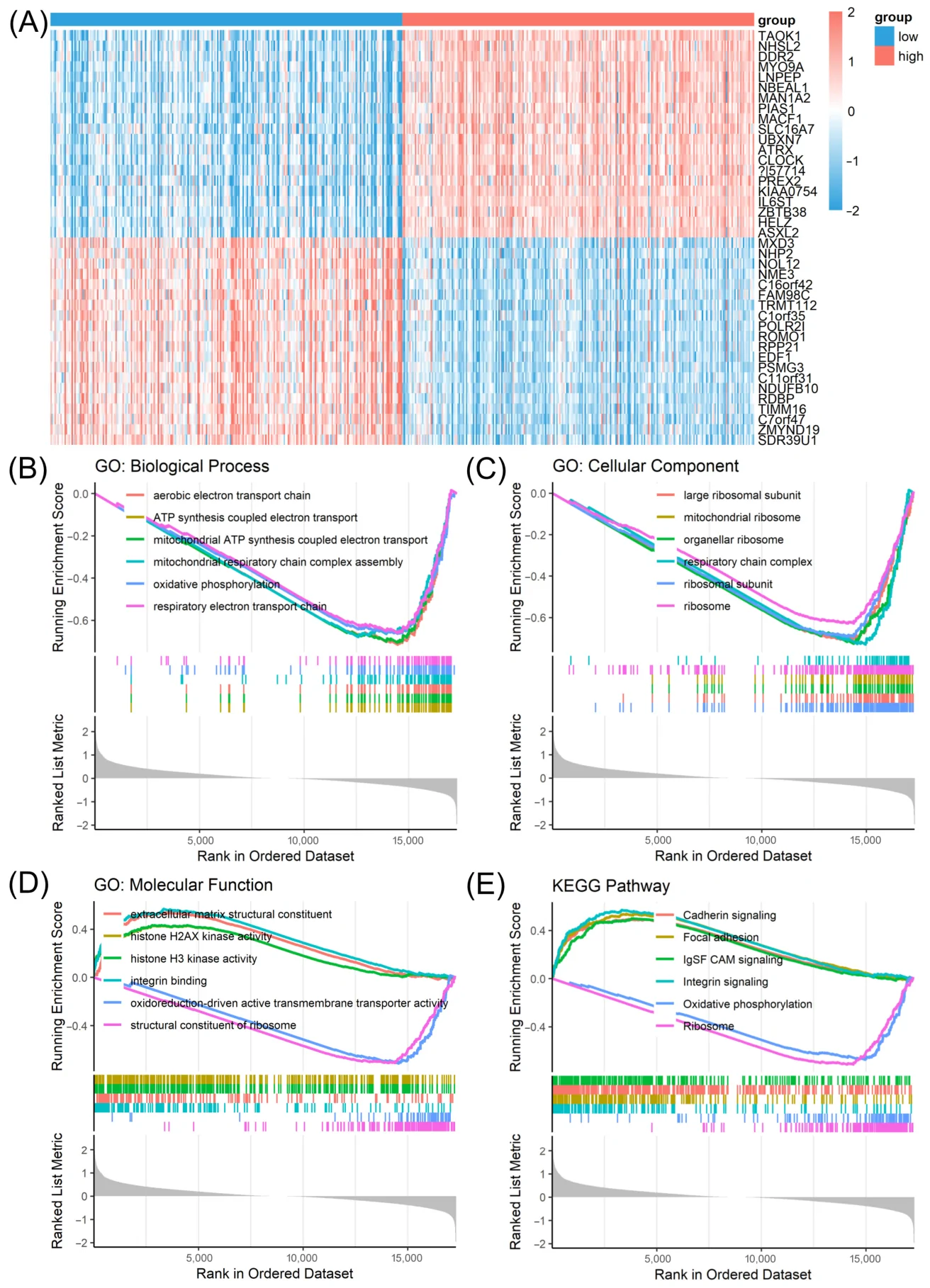
Functional characterization of genes associated with *LPP* expression. (**A**) Heatmap displaying the top upregulated and downregulated DEGs distinguishing high- and low-*LPP* expression groups in The Cancer Genome Atlas Prostate Adenocarcinoma (TCGA-PRAD) cohort. Blue and red column headers denote the low- and high-*LPP* expression groups, respectively. Gene set enrichment plots for genes correlated with *LPP* expression, highlighting key enriched pathways in GO: (**B**) biological processes, (**C**) cellular components, (**D**) molecular functions, and (**E**) KEGG pathways. Positive enrichment scores indicate pathways enriched in the high-*LPP* group, whereas negative scores reflect enrichment in the low-*LPP* group.

To elucidate the downstream molecular consequences of *LPP* dysregulation in prostate cancer, The Cancer Genome Atlas Prostate Adenocarcinoma (TCGA-PRAD) samples were stratified into high- and low-expression groups based on the median transcript levels of *LPP*, and differential expression and GSEA were performed. Heatmap visualization of the top DEGs distinguished the two groups (Figure 7A and Table S3). GSEA of GO biological processes revealed that pathways associated with the aerobic electron transport chain and oxidative phosphorylation were negatively enriched in the high-*LPP* group—equivalently, positively enriched in the low-*LPP* (poor-prognosis) group (Figure 7B). Cellular component analysis demonstrated a concordant negative enrichment of respiratory chain complexes and organellar ribosomes in the high-*LPP* group (Figure 7C). Molecular function analysis identified integrin binding and extracellular matrix (ECM) structural constituents as positively enriched in the high-*LPP* group, whereas structural constituents of ribosome terms were negatively enriched (Figure 7D). KEGG pathway analysis reinforced these findings, with integrin signaling and focal adhesion pathways positively enriched in the high-*LPP* group and ribosome biogenesis and oxidative phosphorylation pathways negatively enriched (Figure 7E). These correlative patterns provide a basis for future studies examining whether *LPP* functionally links DINCH-associated transcriptional changes to prostate cancer progression.

**Figure 8:**
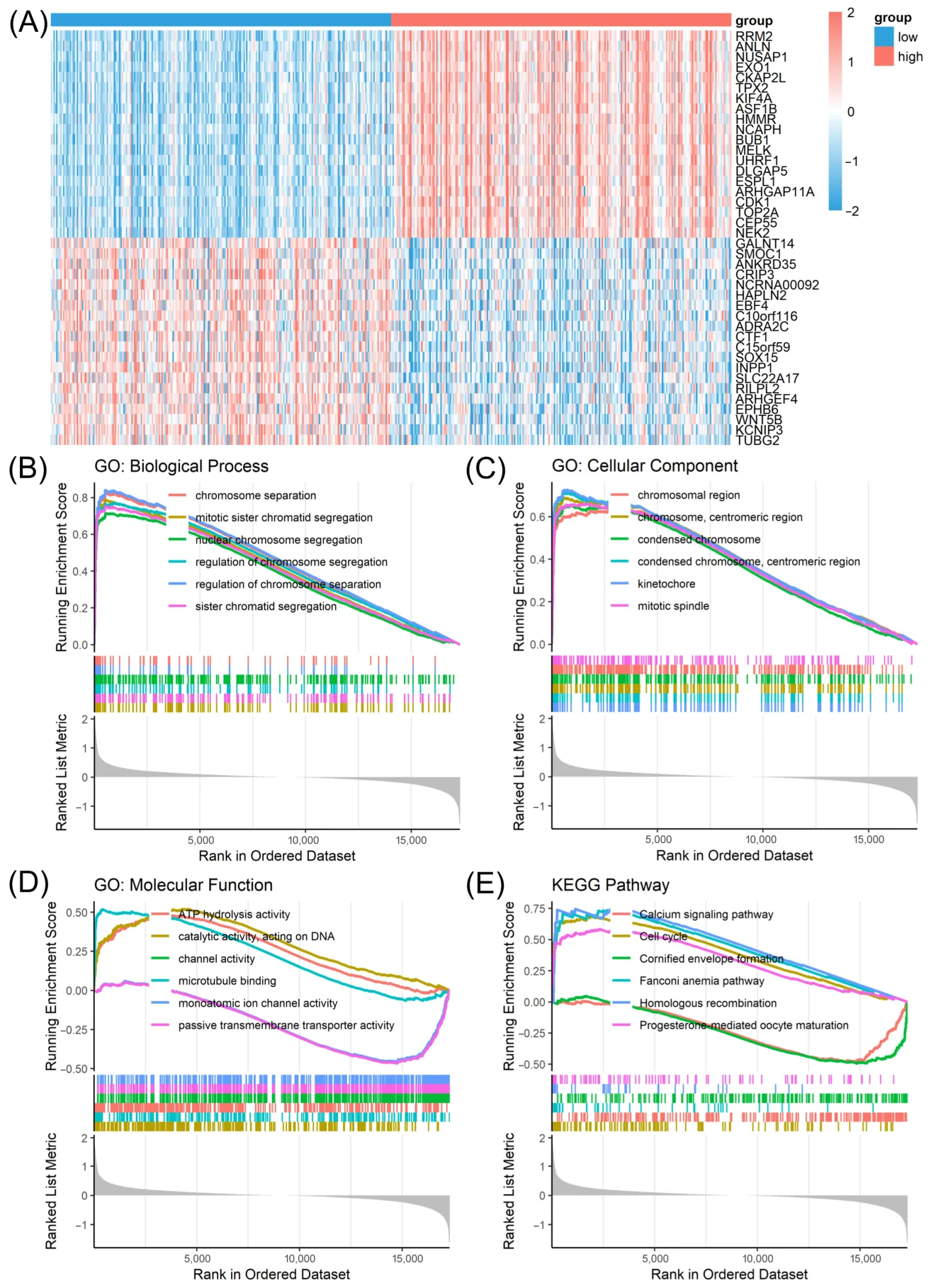
Functional characterization of genes associated with *FAM111B* expression. (**A**) Heatmap displaying the top upregulated and downregulated DEGs distinguishing high- and low-*FAM111B* expression groups in the TCGA-PRAD cohort. Gene set enrichment plots for genes correlated with *FAM111B* expression, highlighting key enriched pathways in GO: (**B**) biological processes, (**C**) cellular components, (**D**) molecular functions, and (**E**) KEGG pathways.

Patient stratification based on *FAM111B* expression revealed a contrasting, hyper-proliferative molecular signature with a distinct transcriptional profile. Heatmap clustering defined a clear transcriptomic segregation between the high- and low-*FAM111B* patient groups (Figure 8A and Table S4). GO biological process analysis showed that regulation of chromosome segregation and mitotic sister chromatid segregation were positively enriched in the high-*FAM111B* group (Figure 8B). Consistent with these mitotic signatures, cellular component analysis revealed positive enrichment of kinetochores and centromeric regions of condensed chromosome terms (Figure 8C). Molecular function analysis identified microtubule binding and catalytic activity acting on DNA as positively enriched terms in the high-*FAM111B* group (Figure 8D). KEGG pathway analysis further highlighted the positive enrichment of homologous recombination and cell cycle pathways in the high-*FAM111B* group (Figure 8E). Taken together, these associations provide biological context for the adverse prognosis associated with elevated *FAM111B* expression but do not establish that *FAM111B* mediates an effect of DINCH on prostate cancer outcomes.

## 3. Discussion

Our study integrated in vitro transcriptomic profiling of DINCH-exposed human prostate cells with clinical genetic association analyses, providing an exploratory toxicogenomic framework to identify candidate plasticizer-responsive genes with potential relevance to prostate cancer progression. Our data showed that exposure to DINCH was associated with extensive transcriptional reprogramming, prominently suppressing cell–matrix and focal adhesion pathways while activating nucleosome assembly programs. Genetic screening prioritized *LPP* and *FAM111B* as key candidates, with germline variants in these genes showing nominal associations with OS and CSS in patients undergoing ADT, although these associations did not meet conventional FDR-adjusted significance thresholds and should therefore be interpreted as suggestive signals requiring independent cohort validation. This exploratory pattern, encompassing repression of *LPP*-associated adhesion pathways and enrichment of *FAM111B*-associated proliferative signatures, suggests that DINCH exposure induces transcriptional alterations in candidate genes that harbor independent prognostic relevance in prostate cancer. However, these findings are correlative and do not establish a causal role for DINCH in promoting aggressive disease or clinical progression. Importantly, unlike our previous studies that evaluated predefined candidate genes in the same clinical cohort, the present work employed an unbiased toxicogenomic pipeline to identify DINCH-responsive genes prior to genetic evaluation, followed by integrative transcriptomic, genetic, and bioinformatic analyses. Accordingly, this study addresses a distinct biological question and represents an independent analytical framework rather than a reanalysis of previously reported candidate-gene associations.

The transcriptional signature induced by DINCH showed notable convergence with that elicited by the traditional phthalate metabolite mono-2-ethylhexyl phthalate (MEHP), suggesting that disruption of cellular adhesion may represent a broader prostate epithelial response to plasticizer exposure. In PNT1A cells, DINCH consistently suppressed cell–substrate and cell–matrix adhesion, focal adhesion, and actin cytoskeleton regulation, while additionally promoting nucleosome assembly and chromatin-associated programs. Similarly, MEHP exposure produced negative enrichment of cell–matrix adhesion, focal adhesion, and cell–cell junction signatures across multiple concentrations [15]. This shared repression of adhesion-related networks supports the possibility that compromised epithelial structural integrity is a general response to chemically distinct plasticizers rather than a DINCH-specific effect. Nevertheless, the prominent chromatin-remodeling signature observed with DINCH was not similarly emphasized following MEHP exposure, which instead showed additional alterations in axon development and cell morphogenesis pathways. These differences suggest that DINCH may engage distinct upstream regulatory mechanisms despite converging on focal adhesion dysfunction.

The transcriptomic profile observed following DINCH exposure, particularly the negative enrichment of focal adhesion, actin cytoskeleton regulation, and cell–matrix adhesion pathways, suggests a coordinated disruption of integrin-dependent signaling networks that maintain epithelial homeostasis. Integrins function as major ECM receptors and transmit mechanical and biochemical signals through focal adhesion complexes to regulate cell adhesion, migration, survival, and gene expression [20]. The concurrent downregulation of LPP, a focal adhesion adaptor protein localized at cell-cell and cell-ECM contacts, further supports impairment of adhesion integrity and mechanosensing [21]. Because focal adhesions are central mechanotransduction hubs, reduced integrin signaling may alter cytoskeletal tension and ECM remodeling, thereby reshaping transcriptional programs that govern epithelial plasticity [22]. Notably, altered ECM stiffness and integrin signaling can activate mechanosensitive pathways that promote epithelial-mesenchymal plasticity, invasion, and cancer progression [23]. Given that DINCH metabolites can activate PPAR signaling [4], it is plausible that DINCH-induced nuclear receptor activation intersects with adhesion-dependent pathways, ultimately contributing to the transcriptomic changes observed in prostate epithelial cells.

Although LPP (LIM domain-containing preferred translocation partner in lipoma) is often characterized as a pro-migratory adaptor protein [24], accumulating evidence highlights its context-dependent tumor-suppressive functions [21,25,26]. Structurally, LPP localizes to focal adhesions and cell–cell contacts, interacting with polarity scaffolds like Scrib to reinforce epithelial integrity and contact-dependent growth inhibition [26]. While its loss has been shown to drive collective invasion and adhesion remodeling in other cancer models [21], its role in prostate cancer has remained underexplored. Consistent with a tumor-suppressive paradigm, our pathway analyses demonstrated positive enrichment of integrin signaling and focal adhesion pathways in the high-*LPP* group, alongside negative enrichment of ribosome biogenesis and oxidative phosphorylation. However, whether *LPP* functionally mediates DINCH-associated effects on prostate cancer progression remains to be determined. At the germline level, *LPP* polymorphisms are established susceptibility signals in various malignancies [27,28,29,30], and our functional annotation of rs1040033 indicates its residence within a region exhibiting weak enhancer chromatin signatures. However, the absence of established prostate-specific expression quantitative trait loci (eQTL) evidence and a lack of direct proof demonstrating that DINCH modulates the penetrance of these variants limits definitive mechanistic interpretation. Consequently, the potential intersection between plasticizer-induced *LPP* repression and host genetic background remains an exploratory hypothesis warranting further investigation into *LPP*’s protective role in the prostate epithelium.

*FAM111B* (FAM111 trypsin-like peptidase B) encodes a nuclear protein that functions as a broadly acting oncogenic driver across multiple human malignancies, including prostate cancer [31]. Recent functional evidence in prostate cancer indicates that *FAM111B* suppresses *ATF3*, reduces *ATF3* occupancy at the *KRAS* promoter, and consequently activates the RAF1–MEK–ERK cascade, thereby attenuating apoptosis and enhancing tumor-cell survival [32]. *FAM111B* has also been shown to promote prostate cancer metastasis by upregulating lactate dehydrogenase A, which enhances glycolytic activity and provides the metabolic capacity required for aggressive tumor-cell behavior [33]. Consistent with these prostate cancer-specific observations, *FAM111B* silencing in ovarian cancer decreases AKT phosphorylation, induces G1/S cell-cycle arrest, and increases p53- and caspase-associated apoptosis, suggesting that *FAM111B* may sustain proliferation through coordinated activation of AKT signaling and suppression of apoptotic pathways [34]. Furthermore, pan-cancer analyses have linked elevated *FAM111B* expression to chromosome instability, DNA-repair programs, accelerated cell-cycle progression, and reduced apoptosis, providing a plausible mechanistic explanation for the homologous-recombination and mitotic signatures observed in our high-*FAM111B* prostate cancer group [35]. Rather than acting through a single tissue-specific pathway, collective experimental evidence across diverse carcinomas demonstrates that *FAM111B* subverts cell cycle checkpoints, suppresses apoptosis, and drives proliferation by intersecting with major oncogenic axes, including the KRAS, p53, and PI3K/AKT signaling pathways [34,36,37,38,39,40]. Consistent with these established pro-survival functions, our pathway analyses demonstrated strong positive enrichment of homologous recombination and cell cycle pathways in the high-*FAM111B* prostate cancer group. However, these correlative data do not demonstrate that *FAM111B* mediates DINCH-related effects on clinical outcomes. Supporting the disease relevance of this locus, large-scale GWAS have identified the *FAM111A*–*FAM111B* region at 11q12 as a genome-wide significant susceptibility locus for prostate cancer predisposition [41]. Furthermore, RegulomeDB annotation of rs7110278 indicates its location within key regulatory regions, and the FIVEx database confirms its association with the expression of the *FAM111B* transcript ENST00000411426 specifically in prostate tissues (*p* = 0.0037). Although dedicated functional assays are required to fully delineate these germline mechanisms, these integrated findings support the potential biological and prognostic relevance of *FAM111B* in advanced prostate cancer biology.

The identification of *LPP* and *FAM111B* should also be interpreted within the rapidly evolving landscape of molecular biomarkers for prostate cancer prognosis and treatment stratification. Recent genomic and transcriptomic studies have demonstrated that integrating molecular biomarkers with conventional clinicopathological variables can improve prognostic accuracy and facilitate precision medicine in advanced prostate cancer. In particular, alterations involving aneuploidy driver genes, immune infiltration, and the tumor microenvironment have emerged as important determinants of disease aggressiveness and therapeutic response [42,43,44]. Although *LPP* and *FAM111B* have not yet been established as clinically validated biomarkers in prostate cancer, our integrative toxicogenomic approach identified these candidate genes through initial in vitro profiling, suggestive germline associations, and multi-cohort transcriptomic analyses. These complementary lines of evidence suggest that *LPP* and *FAM111B* are biologically plausible candidate biomarkers that warrant further investigation in treatment-relevant and clinically annotated prostate cancer models.

The principal strengths of this study include the integration of in vitro transcriptomic profiling with clinical genetic association analyses in a well-characterized cohort of 630 ADT-treated patients, corroborated by large-scale pooled analyses across multiple independent prostate cancer datasets. Crucially, the public clinical datasets evaluate only the baseline clinical and prognostic relevance of *LPP* and *FAM111B* in prostate cancer tissue, and do not provide evidence regarding patient-level DINCH exposure. Several limitations warrant consideration. First, while chosen to model early exposure events, the use of the immortalized non-tumorigenic PNT1A cell line cannot fully recapitulate the complexity of established prostate cancer biology, as it lacks the tumor microenvironment, intratumoral heterogeneity and the selective pressures imposed by androgen deprivation. Consequently, future studies must validate these findings in diverse prostate cancer models under androgen-deprived conditions. Second, although urinary DINCH metabolites are consistently detected in human biomonitoring studies, there is currently insufficient pharmacokinetic evidence linking urinary metabolite concentrations with intracellular DINCH levels in prostate tissue. Therefore, the biological relevance of the in vitro exposure concentrations to human prostate exposure remains uncertain, and caution is warranted when extrapolating these findings to clinical exposure scenarios. Third, candidate DEG selection in the discovery dataset relied on nominal *p*-values (*p* < 0.01) rather than FDR-adjusted thresholds, reflecting the small sample size (four replicates per condition) of the public dataset; thus, this gene set must be interpreted as an exploratory molecular signature subject to potential false-positive selection. Furthermore, the sequential multi-step filtering workflow employed to prioritize candidate genes introduces potential discovery bias, which may increase the likelihood of selecting false-positive candidates by chance. Fourth, genetic screening was conducted at a nominal *p*-value threshold without strict FDR correction. Because the resulting *q*-values (*LPP*: 0.131; *FAM111B*: 0.575) exceed conventional thresholds, the individual SNP signals should not be framed as established prognostic variants but strictly as suggestive markers requiring independent replication. Fifth, we did not perform functional mechanistic experiments, such as gene knockdown, overexpression, transwell migration/invasion assays, proliferation assays, or Western blotting, which are critical to validate the biological roles of *LPP* and *FAM111B*; the current findings remain correlative and bioinformatic in nature. Sixth, because our clinical cohort is exclusively Taiwanese and our SNP tagging relied strictly on Han Chinese LD patterns, these genetic associations may exhibit ethnic specificity, limiting their direct generalizability to non-Asian populations without additional multi-ethnic validation. Seventh, only two DINCH concentrations were evaluated at a single 48 h time point. Although more DEGs were identified at the higher concentration, these findings do not establish a dose–response relationship and may reflect a threshold effect. Further studies using additional concentrations and time points are warranted. Eighth, substantial heterogeneity was observed in the pooled expression analyses. This variability may reflect differences in gene-expression platforms, including distinct microarray and RNA-sequencing approaches, as well as variations in sample processing and cohort characteristics. Because detailed metadata were not consistently available across datasets, the sources of heterogeneity could not be formally investigated. Finally, because patient-level DINCH exposure data and germline genotypes were not measured within the same individuals, this study did not formally test a true gene–environment interaction, and no direct causal relationship between DINCH exposure and clinical prostate cancer progression can be inferred.

## 4. Materials and Methods

### 4.1. Transcriptomic Analysis of DINCH Exposure in Human Prostate Epithelial Cells

To characterize the transcriptional responses elicited by DINCH in prostate epithelial cells, a publicly available gene expression dataset was obtained from the Gene Expression Omnibus (accession number GSE67396). The dataset was generated using the immortalized, non-tumorigenic human prostate epithelial cell line PNT1A to purposefully model baseline transcriptional alterations induced by initial environmental exposure in a normal epithelial context. Cells were maintained under standard culture conditions and treated for 48 h with either a vehicle control (DMSO) or DINCH at a low dose (0.01 μM) or a high dose (1 μM), with four independent biological replicates per treatment group. Total RNA was extracted from the treated cells and subjected to transcriptomic profiling using the Agilent SurePrint G3 Human Gene Expression 8×60K Microarray platform. Processed expression matrices were downloaded, and data normalization and precision weighting were performed using the voom function implemented in the limma package (v3.64.1) in R (v4.5.1; R Foundation for Statistical Computing, Vienna, Austria). Differential expression analyses comparing each DINCH-treated group with the vehicle control were conducted using limma. DEGs were identified using a nominal significance threshold of *p* < 0.01, which was applied strictly as an exploratory screening criterion for candidate-gene prioritization. Given the sample size of four biological replicates per condition, applying a strict FDR threshold severely limited candidate gene yield; therefore, nominal filtering was implemented as an exploratory discovery step. A tiered prioritization strategy was employed to preserve broader biological networks: DEGs were first filtered using the nominal threshold and subsequently prioritized based on concordant directional changes across both the low- and high-dose exposure groups. The intersection of consistently regulated genes across both doses was used to define a candidate gene set for subsequent genetic association and functional analyses. Heatmaps of the DEGs were generated using the pheatmap package (v1.0.13) with Z-score normalization. GSEA was performed using ranked log fold-change values to map altered functional networks [45]. Enrichment profiles were systematically categorized across GO terms, including biological processes, cellular components, and molecular functions, as well as KEGG pathways, using the clusterProfiler package (v4.16.0). Permutation-based enrichment significance was determined using 1000 permutations, with an adjusted *p* < 0.05 considered statistically significant.

### 4.2. Study Population and Clinical Data Collection

Participants were enrolled from an ongoing hospital-based prostate cancer cohort established in Taiwan. Consecutive patients with histologically confirmed prostatic adenocarcinoma were recruited at three tertiary referral centers: National Taiwan University Hospital, Kaohsiung Medical University Hospital, and Kaohsiung Veterans General Hospital. Eligible individuals had initiated primary ADT, consisting of either bilateral orchiectomy or treatment with luteinizing hormone–releasing hormone agonists, with or without concomitant antiandrogen therapy. Patients lacking complete clinicopathological information or adequate follow-up records were excluded from the analysis. The study protocol was approved by the Institutional Review Board of Kaohsiung Medical University Hospital (KMU-HIRB-2013132), and written informed consent was obtained from all participants before study enrollment. Baseline demographic and clinical variables, including age at ADT initiation, baseline PSA level, clinical tumor stage, and Gleason score, were extracted from medical records. The primary clinical endpoints were OS (time from ADT initiation to death from any cause) and CSS (time from ADT initiation to death directly attributable to prostate cancer progression). The cause of death was verified by cross-referencing patients’ medical records with the National Death Registry maintained by Taiwan’s Ministry of Health and Welfare. Over a median follow-up of 165.8 months, 414 deaths were recorded, of which 314 were attributable to prostate cancer (Table S1) [46]. Key clinical variables, including age, PSA level at ADT initiation, clinical stage, and Gleason score, were significantly associated with OS and CSS (*p* < 0.05).

### 4.3. SNP Selection and Genotyping

Candidate genes for germline variant analysis were prioritized based on their concordant dysregulation across both the low- and high-dose DINCH transcriptomic datasets. Among the 83 shared DEGs, 14 genes were excluded because they either lacked SNPs with a minor allele frequency (MAF) > 0.03 in the Han Chinese reference population or were located on the X chromosome or mitochondrial genome. Haplotype-tagged SNPs within the remaining 69 shared DEGs were selected using Haploview software (v4.2) based on linkage disequilibrium (LD) patterns in Han Chinese reference populations (Beijing Han Chinese and Southern Han Chinese) from the 1000 Genomes Project [47], with an LD threshold of *r*^2^ > 0.8. Genomic DNA was isolated from peripheral blood leukocytes using the QIAamp DNA Blood Mini Kit (Qiagen, Venlo, The Netherlands) according to the manufacturer’s instructions. Genotyping was performed using the Affymetrix Axiom platform (Thermo Fisher Scientific, Waltham, MA, USA) at the National Center for Genome Medicine, Taiwan [48]. Quality control filters were applied, excluding individual samples with low genotyping call rates, SNPs with call rates below 90%, a MAF below 0.03, or significant deviations from HWE (*p* < 0.0001). Following quality control, 35 SNPs were excluded because of call rates below 90% and one SNP was excluded because it deviated from HWE, leaving 666 SNPs for subsequent association analyses. Genotyping concordance assessed in duplicate samples was 100%.

### 4.4. Bioinformatic Analyses of LPP and FAM111B Expression and Function

To evaluate the pathological and clinical relevance of *LPP* and *FAM111B* expression in prostate cancer, large-scale transcriptomic data were retrieved from multiple public repositories, including PCaDB [49], the Gene Expression Database of Normal and Tumor Tissues 2 [50], and TCGA-PRAD dataset. These public cohorts were analyzed to establish general tumor-versus-normal expression profiles and baseline prognostic associations in prostate cancer; they do not contain toxicological exposure data and therefore do not assess patient-level DINCH exposure. Differences in the expression levels of *LPP* and *FAM111B* between primary prostate cancer tissues and normal prostate tissues were assessed by pooled analysis using a random-effects model implemented in Review Manager (v5.4.1; Cochrane Collaboration, London, UK), with SMD and corresponding 95% CIs reported as effect size measures. Prognostic relevance was assessed by pooled survival analysis, with HRs and 95% CIs derived from individual study data using an inverse-variance-weighted random-effects model. For downstream mechanistic characterization, TCGA-PRAD patient samples were stratified into high and low-expression groups based on the median transcript levels of *LPP* or *FAM111B*. Differential gene expression analysis between the stratified groups was performed using the limma package. The resulting ranked gene lists were subjected to GSEA against molecular signatures from the GO and KEGG databases to identify significantly enriched biological pathways using the clusterProfiler package. Key enriched pathways were visualized using the enrichplot package (v1.28.2).

### 4.5. Statistical Analyses

Statistical analyses were performed using R and SPSS (v19.0; IBM Corp., Armonk, NY, USA). Associations between germline genetic variants and clinical outcomes (OS and CSS) were evaluated using Cox proportional hazards regression models, with HRs and 95% CIs estimated for each SNP using an additive genetic model. For this exploratory genetic association study, initial screening was conducted at a nominal two-sided *p*-value threshold of *p* < 0.05 to avoid the premature exclusion of potentially relevant biological signals. To account for multiple comparisons, FDR *q*-values were estimated using the Storey method for all tested SNPs. Candidate genes were subsequently prioritized based on convergent statistical evidence across both survival endpoints as an exploratory heuristic rather than formal statistical validation. Between-group differences in the relative mRNA expression of candidate genes were evaluated using one-way analysis of variance, and heterogeneity in the pooled analyses was quantified using the *I*^2^ statistic.

## 5. Conclusions

Our findings demonstrate that in vitro DINCH exposure alters the expression of candidate genes, including *LPP* and *FAM111B*, which are independently associated with cell–matrix adhesion, cell-cycle pathways, and clinical survival in prostate cancer cohorts. This study underscores the importance of evaluating the oncological safety of replacement plasticizers and provides exploratory toxicogenomic candidates for future research. Because environmental exposure and clinical outcomes were evaluated across independent experimental and patient datasets without paired personal exposure measurements, these results should not be interpreted as establishing a clinical or causal link between DINCH exposure and prostate cancer progression. Prospective studies incorporating paired biomonitoring exposure measurements, germline genetics, and longitudinal clinical data, together with functional mechanistic investigations of *LPP* and *FAM111B* in experimental prostate cancer models, are warranted to clarify their biological roles and potential clinical relevance.

## Data Availability

Due to privacy and ethical restrictions, the data presented in this study are not publicly available but can be obtained from the corresponding authors upon reasonable request.

## Supplementary Materials

The following supporting information can be downloaded at https://www.mdpi.com/article/10.3390/ijms27177686/s1.
