# Supplementary material for: Exploratory Toxicogenomic Profiling Identifies Candidate DINCH-Responsive Genes Relevant to Prostate Cancer
Source: Int J Mol Sci. 2026 Aug 27;27(17):7686. doi: 10.3390/ijms27177686 (PMC13566863; doi:10.3390/ijms27177686)
Supplement: Supplementary file 1 [file ijms-27-07686-s001.zip › ijms-4390345-supplementary.pdf]

Table S1. Clinical and pathological characteristics of the study population

Characteristics	n (%)	OS		CSS	
		HR (95% CI)	*p*	HR (95% CI)	*p*
Total, n (%)	630	414 (65.7)		314 (49.8)	
Age at diagnosis, years					
Median (IQR)	73 (67-79)				
<74	344 (54.7)	1.00		1.00	
≥74	285 (45.3)	1.67 (1.37-2.03)	<0.001	1.32 (1.06-1.65)	0.014
PSA at ADT initiation, ng/mL					
Median (IQR)	34.5 (11.25-129)				
<35	307 (50.6)	1.00		1.00	
≥35	300 (49.4)	1.99 (1.63-2.44)	<0.001	2.31 (1.83-2.91)	<0.001
Clinical stage at diagnosis					
T1/T2	187 (29.9)	1.00		1.00	
T3/T4/N1	205 (32.8)	1.07 (0.82-1.39)	0.642	1.08 (0.79-1.49)	0.636
M1	233 (37.3)	2.51 (1.97-3.19)	<0.001	3.10 (2.33-4.11)	<0.001
Gleason score at diagnosis					
2-6	188 (30.6)	1.00		1.00	
7	194 (31.6)	1.06 (0.82-1.38)	0.640	1.06 (0.78-1.44)	0.694
8-10	232 (37.8)	1.96 (1.54-2.48)	<0.001	2.14 (1.63-2.82)	<0.001

Abbreviations: OS, overall survival; CSS, cancer-specific survival; HR, hazard ratio; CI, confidence interval; IQR, interquartile range; PSA, prostate-specific antigen; ADT, androgen deprivation therapy.

Median follow-up duration was 165.8 months.

Totals may not sum to the overall number of patients because of missing data.

Table S2. List of differentially expressed genes induced by low- and high-dose DINCH exposure in PNT1A prostate epithelial cells

Gene	Low						High					
	logFC	AveExpr	t	P.Value	adj.P.Val	B	logFC	AveExpr	t	P.Value	adj.P.Val	B
NNMT	0.3221	14.6056	3.5601	0.0032	0.8207	-2.9198	0.2966	14.6056	3.2781	0.0056	0.2894	-2.0375
PXK	0.3358	8.2253	3.7411	0.0022	0.8207	-2.7984	0.3526	8.2253	3.9284	0.0015	0.2312	-0.9439
NPY	0.4825	8.1315	3.0792	0.0083	0.8207	-3.2591	0.5198	8.1315	3.3171	0.0052	0.2868	-1.9716
CDKN2A-AS1	0.4523	5.4130	3.5019	0.0036	0.8207	-2.9596	0.5420	5.4130	4.1963	0.0009	0.2309	-0.5031
100131490	0.4660	5.3675	5.2142	0.0001	0.6585	-1.9636	0.3489	5.3675	3.9045	0.0016	0.2312	-0.9835
CCDC169	0.4220	5.4938	4.5723	0.0004	0.8207	-2.2932	0.4424	5.4938	4.7935	0.0003	0.1736	0.4454
MTRFR	0.2689	10.9285	3.0858	0.0081	0.8207	-3.2544	0.2658	10.9285	3.0503	0.0087	0.3179	-2.4230
SRP72	0.2682	11.0486	3.5398	0.0033	0.8207	-2.9337	0.2554	11.0486	3.3715	0.0046	0.2835	-1.8794
EPS8	0.4086	9.7961	4.3697	0.0007	0.8207	-2.4082	0.2813	9.7961	3.0084	0.0095	0.3224	-2.4937
ATRNL1	0.2702	5.8508	3.2573	0.0058	0.8207	-3.1309	0.2724	5.8508	3.2827	0.0055	0.2877	-2.0297
LINC01341	0.3899	5.6658	4.6311	0.0004	0.8207	-2.2609	0.2768	5.6658	3.2884	0.0055	0.2868	-2.0202
RDH14	0.2501	9.8150	3.5610	0.0032	0.8207	-2.9192	0.2175	9.8150	3.0966	0.0080	0.3049	-2.3448
HOXA-AS3	0.3347	5.8649	3.6737	0.0025	0.8207	-2.8432	0.2876	5.8649	3.1565	0.0071	0.2999	-2.2434
PROSER2-AS1	0.3951	5.2380	4.1014	0.0011	0.8207	-2.5686	0.3865	5.2380	4.0124	0.0013	0.2312	-0.8048
MRE11	0.2864	9.6766	3.6117	0.0029	0.8207	-2.8848	0.3062	9.6766	3.8619	0.0018	0.2312	-1.0545
440330	0.3081	5.5103	3.7237	0.0023	0.8207	-2.8099	0.2780	5.5103	3.3602	0.0047	0.2835	-1.8985
AGO1	0.2426	9.3176	3.3745	0.0046	0.8207	-3.0481	0.3580	9.3176	4.9797	0.0002	0.1684	0.7299
ZNF680	0.2606	5.5239	3.1785	0.0068	0.8207	-3.1873	0.3116	5.5239	3.8004	0.0020	0.2387	-1.1570
ADAD2	0.2579	6.2477	3.1090	0.0078	0.8207	-3.2375	0.4057	6.2477	4.8914	0.0002	0.1718	0.5957
LOC401557	0.5670	6.8064	3.3426	0.0049	0.8207	-3.0705	0.8174	6.8064	4.8186	0.0003	0.1718	0.4841
SNAR-C4	0.6018	6.5429	3.7788	0.0021	0.8207	-2.7736	0.6839	6.5429	4.2939	0.0008	0.2271	-0.3447
RNA18SN5	1.1100	13.5229	3.8834	0.0017	0.8207	-2.7057	1.4542	13.5229	5.0878	0.0002	0.1591	0.8925

CYGB	0.7469	13.4452	4.0722	0.0012	0.8207	-2.5866	1.0450	13.4452	5.6973	0.0001	0.0887	1.7708
OTX1	0.2870	6.1004	3.0667	0.0085	0.8207	-3.2682	0.3267	6.1004	3.4909	0.0037	0.2713	-1.6774
CCDC71L	0.4519	10.6790	3.9517	0.0015	0.8207	-2.6621	0.4711	10.6790	4.1190	0.0011	0.2312	-0.6296
GTF2A1	0.3398	9.9592	3.3856	0.0045	0.8207	-3.0403	0.3507	9.9592	3.4947	0.0036	0.2713	-1.6710
BTN2A1	0.3871	8.3073	4.1653	0.0010	0.8207	-2.5296	0.4197	8.3073	4.5167	0.0005	0.1956	0.0121
TMEFF2	0.3442	7.7405	3.3366	0.0050	0.8207	-3.0748	0.3089	7.7405	2.9941	0.0098	0.3225	-2.5178
NDUFB6	0.3272	12.1118	3.2847	0.0055	0.8207	-3.1114	0.3750	12.1118	3.7637	0.0021	0.2425	-1.2184
CD86	1.0676	6.8438	3.7729	0.0021	0.8207	-2.7775	0.8936	6.8438	3.1578	0.0071	0.2999	-2.2413
BCL2A1	0.2881	9.7990	3.3669	0.0047	0.8207	-3.0534	0.3047	9.7990	3.5609	0.0032	0.2590	-1.5592
DNAL1	0.3265	9.6890	3.3274	0.0051	0.8207	-3.0812	0.3853	9.6890	3.9268	0.0016	0.2312	-0.9465
ABO	0.4992	6.9844	3.2446	0.0060	0.8207	-3.1399	0.6798	6.9844	4.4181	0.0006	0.2039	-0.1450
HOXA6	0.6091	7.8715	3.8140	0.0019	0.8207	-2.7506	0.6668	7.8715	4.1752	0.0010	0.2312	-0.5376
LARS1	0.2513	10.2194	3.0288	0.0091	0.8207	-3.2959	0.2819	10.2194	3.3970	0.0044	0.2822	-1.8362
RIT1	0.7223	7.3021	5.1256	0.0002	0.6585	-2.0061	0.6344	7.3021	4.5013	0.0005	0.1956	-0.0123
PATZ1	0.3190	6.9039	3.2355	0.0061	0.8207	-3.1465	0.3278	6.9039	3.3250	0.0051	0.2868	-1.9582
CSDE1	0.4179	11.7144	4.4212	0.0006	0.8207	-2.3785	0.3975	11.7144	4.2053	0.0009	0.2309	-0.4885
KRTAP4-11	0.5777	8.4380	3.9511	0.0015	0.8207	-2.6625	0.9203	8.4380	6.2938	0.0000	0.0887	2.5661
PBLD	0.3539	7.0996	3.8309	0.0019	0.8207	-2.7397	0.5317	7.0996	5.7556	0.0001	0.0887	1.8514
SHISA7	0.3031	6.3431	3.7393	0.0022	0.8207	-2.7996	0.4560	6.3431	5.6266	0.0001	0.0895	1.6723
DLGAP4	0.4196	9.5449	3.3669	0.0047	0.8207	-3.0534	0.4453	9.5449	3.5733	0.0031	0.2585	-1.5383
H4C12	0.9032	12.6869	4.6794	0.0004	0.8207	-2.2346	1.0657	12.6869	5.5209	0.0001	0.0924	1.5234
H4C11	1.0934	11.6907	5.2856	0.0001	0.6585	-1.9301	1.1879	11.6907	5.7420	0.0001	0.0887	1.8327
TRPV5	0.2699	5.6397	3.0338	0.0090	0.8207	-3.2923	0.3235	5.6397	3.6371	0.0027	0.2517	-1.4308
COA7	0.2916	9.8730	3.8170	0.0019	0.8207	-2.7486	0.2382	9.8730	3.1182	0.0076	0.3032	-2.3083
UGT2B10	0.3213	6.1121	3.1850	0.0067	0.8207	-3.1826	0.3783	6.1121	3.7500	0.0022	0.2448	-1.2413

FAM111B	0.5614	10.3889	5.4502	0.0001	0.6585	-1.8551	0.4702	10.3889	4.5645	0.0005	0.1956	0.0879
DDAH2	0.2724	11.2653	3.0328	0.0090	0.8207	-3.2930	0.2891	11.2653	3.2184	0.0063	0.2936	-2.1387
MROH7	0.4787	6.1702	4.1374	0.0010	0.8207	-2.5465	0.5524	6.1702	4.7741	0.0003	0.1739	0.4154
DPY19L2	-0.6450	5.8762	-3.4821	0.0037	0.8207	-2.9733	-0.7314	5.8762	-3.9482	0.0015	0.2312	-0.9110
TAMALIN	-0.2837	6.7318	-3.2639	0.0057	0.8207	-3.1262	-0.2881	6.7318	-3.3152	0.0052	0.2868	-1.9748
PDE5A	-0.3209	9.3949	-3.1139	0.0077	0.8207	-3.2340	-0.3556	9.3949	-3.4501	0.0040	0.2713	-1.7464
CIRBP	-0.3383	9.2149	-3.0975	0.0080	0.8207	-3.2459	-0.3265	9.2149	-2.9892	0.0099	0.3225	-2.5261
PPL	-0.6161	7.5966	-3.4955	0.0036	0.8207	-2.9640	-0.6231	7.5966	-3.5356	0.0033	0.2651	-1.6020
DPAGT1	-0.3376	7.9867	-3.1567	0.0071	0.8207	-3.2030	-0.3546	7.9867	-3.3151	0.0052	0.2868	-1.9748
MLF2	-0.3037	11.7186	-3.0291	0.0091	0.8207	-3.2957	-0.3205	11.7186	-3.1965	0.0065	0.2995	-2.1757
WNK2	-0.2175	5.9234	-3.0727	0.0084	0.8207	-3.2639	-0.2169	5.9234	-3.0646	0.0085	0.3127	-2.3988
COPZ1	-0.2122	12.3383	-3.1769	0.0068	0.8207	-3.1884	-0.2586	12.3383	-3.8716	0.0017	0.2312	-1.0383
STRBP	-0.3030	10.8622	-3.5370	0.0033	0.8207	-2.9356	-0.3188	10.8622	-3.7210	0.0023	0.2448	-1.2899
MYO1D	-0.2897	6.2417	-3.3806	0.0045	0.8207	-3.0438	-0.3300	6.2417	-3.8512	0.0018	0.2314	-1.0723
EPPK1	-0.4544	6.7270	-3.4817	0.0037	0.8207	-2.9736	-0.5945	6.7270	-4.5556	0.0005	0.1956	0.0738
GABRE	-0.4308	9.7808	-3.3374	0.0050	0.8207	-3.0741	-0.4617	9.7808	-3.5767	0.0031	0.2580	-1.5326
SF1	-0.2735	9.6052	-3.0976	0.0080	0.8207	-3.2458	-0.3289	9.6052	-3.7260	0.0023	0.2448	-1.2816
348738	-0.2496	5.7933	-3.0024	0.0096	0.8207	-3.3153	-0.3100	5.7933	-3.7284	0.0023	0.2448	-1.2775
LPP	-0.3059	8.8781	-3.1122	0.0077	0.8207	-3.2351	-0.3379	8.8781	-3.4377	0.0041	0.2731	-1.7674
RBM14	-0.3599	8.0597	-4.1859	0.0009	0.8207	-2.5171	-0.3264	8.0597	-3.7965	0.0020	0.2387	-1.1635
PRSS35	-0.4700	5.5195	-4.1775	0.0010	0.8207	-2.5221	-0.4620	5.5195	-4.1059	0.0011	0.2312	-0.6511
SPACA6	-0.6600	7.4193	-3.5020	0.0036	0.8207	-2.9596	-0.7387	7.4193	-3.9199	0.0016	0.2312	-0.9581
DENND3	-0.2424	7.1081	-3.1763	0.0068	0.8207	-3.1889	-0.2614	7.1081	-3.4250	0.0042	0.2766	-1.7889
SNHG20	-0.4767	6.1991	-3.1311	0.0075	0.8207	-3.2215	-0.5167	6.1991	-3.3941	0.0044	0.2822	-1.8411
THBD	-0.2966	7.7958	-3.1405	0.0073	0.8207	-3.2147	-0.3363	7.7958	-3.5602	0.0032	0.2590	-1.5604

CKB	-0.5476	8.4710	-4.1650	0.0010	0.8207	-2.5297	-0.4793	8.4710	-3.6452	0.0027	0.2517	-1.4173
SAMD10	-0.3560	8.0849	-3.8514	0.0018	0.8207	-2.7263	-0.2814	8.0849	-3.0441	0.0088	0.3190	-2.4334
NDRG1	-0.3813	12.1648	-3.1761	0.0068	0.8207	-3.1890	-0.3839	12.1648	-3.1978	0.0065	0.2995	-2.1736
TMEM132E	-0.4190	5.5510	-3.8130	0.0019	0.8207	-2.7513	-0.4545	5.5510	-4.1360	0.0010	0.2312	-0.6016
MT-ND4	-0.7274	11.7283	-3.4902	0.0037	0.8207	-2.9677	-0.6394	11.7283	-3.0677	0.0084	0.3127	-2.3936
SAPCD1	-0.4064	7.1273	-3.6747	0.0025	0.8207	-2.8425	-0.3617	7.1273	-3.2704	0.0057	0.2899	-2.0506
MT-CO3	-0.7412	14.1323	-3.7485	0.0022	0.8207	-2.7935	-0.7387	14.1323	-3.7357	0.0023	0.2448	-1.2653
MT-CYB	-0.6351	13.2812	-3.2184	0.0063	0.8207	-3.1587	-0.6516	13.2812	-3.3021	0.0053	0.2868	-1.9969
57399	-0.3907	5.0329	-4.3565	0.0007	0.8207	-2.4159	-0.3516	5.0329	-3.9208	0.0016	0.2312	-0.9565
CLCN2	-0.2496	6.3414	-3.3312	0.0050	0.8207	-3.0786	-0.3074	6.3414	-4.1027	0.0011	0.2312	-0.6562
ICOSLG	-0.3030	7.4401	-3.0278	0.0091	0.8207	-3.2966	-0.3129	7.4401	-3.1268	0.0075	0.3023	-2.2937

Abbreviations: logFC, log fold change; AveExpr, average expression; t, t-statistic; P.Value, raw *p*-value; adj.P.Val, adjusted *p*-value; B, B-statistic

Table S3. List of top differentially expressed genes distinguishing high- and low-*LPP* expression groups in the TCGA-PRAD prostate cancer cohort

Gene	logFC	AveExpr	t	P.Value	adj.P.Val	B
TAOK1	2.1098	7.3596	21.2769	6.64E-72	6.81E-68	153.0164
NHSL2	1.6325	4.5462	20.7087	3.75E-69	2.57E-65	146.7256
DDR2	2.2127	6.5258	20.6316	8.85E-69	4.54E-65	145.8733
MYO9A	1.5602	8.0978	20.1345	2.24E-66	9.19E-63	140.3805
LNPEP	1.4687	7.4683	19.6007	8.39E-64	2.87E-60	134.4971
NBEAL1	1.8140	6.3766	19.5788	1.07E-63	3.14E-60	134.2560
MAN1A2	0.9684	9.2281	19.3799	9.68E-63	2.48E-59	132.0689
PIAS1	0.7525	8.4539	18.9895	7.24E-61	1.65E-57	127.7852
MACF1	0.7581	12.0271	18.9490	1.13E-60	2.32E-57	127.3413
SLC16A7	1.4629	3.1237	18.8931	2.10E-60	3.91E-57	126.7299
UBXN7	1.2008	7.8497	18.8674	2.78E-60	4.46E-57	126.4477
ATRX	0.7662	10.7255	18.8661	2.82E-60	4.46E-57	126.4341
CLOCK	1.5217	7.7485	18.8206	4.66E-60	6.84E-57	125.9356
?|57714	1.3740	7.7847	18.6524	2.97E-59	4.07E-56	124.0975
PREX2	1.9523	4.4467	18.6331	3.67E-59	4.71E-56	123.8865
KIAA0754	2.2539	6.3310	18.4941	1.69E-58	1.93E-55	122.3689
IL6ST	2.4340	8.9005	18.4059	4.46E-58	4.82E-55	121.4071
ZBTB38	0.8374	10.2955	18.3777	6.09E-58	5.95E-55	121.0996
HELZ	0.6540	9.8718	18.2157	3.60E-57	3.36E-54	119.3356
ASXL2	1.7671	8.0593	18.1698	5.95E-57	5.31E-54	118.8365
MXD3	-1.0681	7.2501	-16.9798	2.47E-51	6.19E-49	105.9934
NHP2	-0.6029	10.7049	-16.9802	2.46E-51	6.19E-49	105.9974

NOL12	-0.6770	8.4080	-16.9837	2.37E-51	6.08E-49	106.0349
NME3	-0.9195	10.8019	-16.9884	2.25E-51	5.86E-49	106.0848
C16orf42	-0.6661	10.1457	-17.0937	7.23E-52	2.03E-49	107.2131
FAM98C	-0.6245	8.7162	-17.0945	7.17E-52	2.03E-49	107.2214
TRMT112	-0.5541	11.2513	-17.1293	4.92E-52	1.44E-49	107.5946
C1orf35	-0.7506	8.5241	-17.1639	3.39E-52	1.02E-49	107.9662
POLR2I	-0.7995	9.2197	-17.1828	2.76E-52	8.60E-50	108.1697
ROMO1	-0.9582	10.0926	-17.1888	2.59E-52	8.41E-50	108.2341
RPP21	-0.7441	8.8316	-17.3351	5.31E-53	1.88E-50	109.8064
EDF1	-0.7345	12.7734	-17.4543	1.46E-53	5.43E-51	111.0905
PSMG3	-0.8409	9.1468	-17.5597	4.63E-54	1.87E-51	112.2265
C11orf31	-0.6361	10.2554	-17.6405	1.92E-54	8.23E-52	113.0992
NDUFB10	-0.6900	11.0677	-17.6590	1.57E-54	6.87E-52	113.2991
RDBP	-0.5321	10.3541	-17.7706	4.66E-55	2.34E-52	114.5061
TIMM16	-1.0317	7.9879	-17.7890	3.82E-55	1.96E-52	114.7053
C7orf47	-0.9858	8.2048	-17.9576	6.06E-56	3.89E-53	116.5324
ZMYND19	-0.7216	8.9218	-18.3801	5.92E-58	5.95E-55	121.1263
SDR39U1	-0.5970	9.8635	-18.5586	8.34E-59	1.01E-55	123.0723

Abbreviations: logFC, log fold change; AveExpr, average expression; t, t-statistic; P.Value, raw *p*-value; adj.P.Val, adjusted *p*-value; B, B-statistic

Table S4. List of top differentially expressed genes distinguishing high- and low-*FAM111B* expression groups in the TCGA-PRAD prostate cancer cohort

Gene	logFC	AveExpr	t	P.Value	adj.P.Val	B
RRM2	1.7826	7.0571	20.5756	1.65E-68	1.69E-64	145.1753
ANLN	1.7311	5.9923	19.6766	3.61E-64	2.47E-60	135.2699
NUSAP1	1.5860	6.9267	19.2864	2.72E-62	1.40E-58	130.9848
EXO1	1.8016	4.1814	19.2451	4.30E-62	1.76E-58	130.5323
CKAP2L	1.7985	3.8856	19.0368	4.29E-61	1.47E-57	128.2506
TPX2	1.6063	7.2141	18.9261	1.46E-60	4.27E-57	127.0398
KIF4A	1.9207	5.8212	18.8274	4.33E-60	1.11E-56	125.9604
ASF1B	1.4234	6.3549	18.8145	4.99E-60	1.14E-56	125.8195
HMMR	1.7966	5.7179	18.7652	8.58E-60	1.76E-56	125.2817
NCAPH	1.6774	5.2320	18.6709	2.42E-59	4.52E-56	124.2525
BUB1	1.8160	5.5729	18.6141	4.53E-59	7.75E-56	123.6327
MELK	1.8065	5.2247	18.5247	1.21E-58	1.91E-55	122.6582
UHRF1	1.5176	5.4657	18.3993	4.80E-58	7.04E-55	121.2928
DLGAP5	1.9181	4.8892	18.3304	1.02E-57	1.40E-54	120.5432
ESPL1	1.6339	4.9340	18.1728	5.75E-57	7.38E-54	118.8310
ARHGAP11A	1.1837	6.2554	18.1392	8.31E-57	1.00E-53	118.4656
CDK1	1.5228	6.9363	18.0803	1.58E-56	1.81E-53	117.8269
TOP2A	1.7660	7.9195	18.0553	2.08E-56	2.25E-53	117.5557
CEP55	1.5748	5.1779	18.0090	3.45E-56	3.54E-53	117.0539
NEK2	1.8589	4.9178	17.9858	4.45E-56	4.35E-53	116.8019
GALNT14	-0.9936	5.2363	-9.2447	6.92E-19	6.55E-17	32.0310
SMOC1	-1.3260	8.2120	-9.2953	4.62E-19	4.41E-17	32.4294

ANKRD35	-0.8174	6.5673	-9.3213	3.75E-19	3.65E-17	32.6346
CRIP3	-1.2046	3.3307	-9.3416	3.18E-19	3.13E-17	32.7958
NCRNA00092	-0.8003	4.9456	-9.3463	3.07E-19	3.04E-17	32.8325
HAPLN2	-0.9960	3.9074	-9.3608	2.73E-19	2.72E-17	32.9479
EBF4	-0.6780	9.3715	-9.4670	1.16E-19	1.18E-17	33.7932
C10orf116	-1.2924	10.8901	-9.7289	1.36E-20	1.43E-18	35.9063
ADRA2C	-1.0258	6.7902	-9.9928	1.51E-21	1.73E-19	38.0750
CTF1	-0.8342	6.6918	-10.0020	1.40E-21	1.61E-19	38.1513
C15orf59	-0.8295	6.2736	-10.0039	1.37E-21	1.59E-19	38.1675
SOX15	-0.8123	5.6036	-10.1029	5.95E-22	7.07E-20	38.9915
INPP1	-0.6642	7.5335	-10.1077	5.72E-22	6.86E-20	39.0317
SLC22A17	-0.7044	9.9881	-10.2870	1.24E-22	1.57E-20	40.5382
RILPL2	-0.4951	9.0310	-10.3515	7.14E-23	9.11E-21	41.0849
ARHGEF4	-0.6250	7.8496	-10.3542	6.98E-23	8.95E-21	41.1079
EPHB6	-1.0033	9.6602	-10.5375	1.43E-23	1.87E-21	42.6720
WNT5B	-0.7401	9.8015	-11.0933	1.06E-25	1.48E-23	47.5197
KCNIP3	-0.7722	6.3522	-11.1434	6.76E-26	9.50E-24	47.9640
TUBG2	-0.5187	8.0057	-11.1720	5.23E-26	7.45E-24	48.2179

Abbreviations: logFC, log fold change; AveExpr, average expression; t, t-statistic; P.Value, raw *p*-value; adj.P.Val, adjusted *p*-value; B, B-statistic
